# Signal transducer and activator of transcription 3 (*STAT3*) promoter methylation and expression in pituitary adenoma

**DOI:** 10.1186/s12881-017-0434-3

**Published:** 2017-07-14

**Authors:** Indre Valiulyte, Giedrius Steponaitis, Daina Skiriute, Arimantas Tamasauskas, Paulina Vaitkiene

**Affiliations:** Laboratory of Neurooncology and Genetics, Neuroscience Institute, Lithuanian University of Health Sciences, Eiveniu str. 2, LT-50009 Kaunas, Lithuania

**Keywords:** Pituitary adenoma, Signal transducer and activator of transcription 3, DNA methylation, mRNA expression, Invasiveness

## Abstract

**Background:**

Pituitary adenoma (PA) is a benign brain tumor that can cause neurological, endocrinological and ophthalmological aberrations. Till now there is a need to identify factors that can influence the tumor invasiveness and recurrence. The aim of this study was to evaluate the associations between the signal transducer and activator of transcription 3 (*STAT3*) promoter methylation, mRNA expression and the invasiveness or recurrence of PAs and patient clinical characteristics.

**Methods:**

Study participants comprised of 102 subjects with a diagnosis of PA: 54 functioning and 48 non-functioning, 58 invasive and 30 non-invasive PAs and 14 relapses. The bisulfite treatment of tumor DNA and methylation-specific polymerase chain reaction (MS-PCR) method was used to determine the *STAT3* gene promoter methylation. For the *STAT3* mRNA expression, the first-strand cDNA was produced from total RNA by using reverse transcriptase and quantitative real-time PCR (qRT-PCR) was performed.

**Results:**

In 10.78% (11/102) of PA tissues *STAT3* gene promoter was methylated. A gender of male and patient group older than 60 years were significantly associated with reduced *STAT3* mRNA expression (Mann-Whitney test, *p* = 0.025, *p* = 0.047, respectively). However, no more statistical differences were found between *STAT3* promoter methylation, mRNA expression and patient clinical characteristics or PA invasiveness or recurrence.

**Conclusions:**

Further investigations are needed to clarify the influence of *STAT3* gene promoter methylation and mRNA expression changes in PAs.

## Background

Pituitary adenomas (PA) arise from adenohypophyseal cells and account for 10–15% of all intracranial neoplasms. About 0.2% of all PAs with subarachnoid, brain or systemic metastasis are considered to be malignant [1]. Historically, PA tumors have been classified according to size as microadenomas (less than 1 cm) or macroadenomas (greater than or equal to 1 cm). Depending on PAs hormonal activity, they are also classified as either functional - secreting adrenocorticotropic hormone (ACTH), growth hormone (GH), prolactin (PRL), thyroid-stimulating hormone (TSH), luteinizing hormone (LH), and follicle-stimulating hormone (FSH) or non-functional - do not cause clinical hormone hypersecretion [2].

Surgical resection is the first-line of treatment for PAs, except for PRL-producing adenomas. Residual or recurrence tumors require re-operation, medical treatment or radiation [3]. However, the prediction of pituitary tumor behavior remains a challenge and factors known to be involved in pituitary tumorigenesis are not predictive of invasiveness or aggressiveness [4, 5]. Therefore, a better understanding of PA tumorigenesis is important for the development of novel targeted drugs.

In many cancers, including PAs, epigenetic changes have been involved in development of tumors [6]. Pituitary tumorigenesis frequently involves mutations of oncogenes (*Cyclin D1*, *C-myc*), tumor suppressor genes (*MEN1*, *p53*), cell cycle regulator genes (*AIP*, *PRKAR1A*), and epigenetic factors (DNA methylation, imprinting, histone modification) [2, 6]. For example, the methylation and deletion of *DAP-kinase* gene was associated with highly invasive and/or metastatic pituitary tumors [7]. Also, the methylation of the growth arrest and DNA damage-inducible gene (*GADD45G*) leads to gene silencing and is associated with the development of somatotropinomas, prolactinomas, and non-functioning adenomas [8].

One of the potential marker for PA diagnosis and prognosis could be signal transducer and activator of transcription 3 (*STAT3*) gene, a member of the STAT family of proteins that mediates cytokine signaling and nuclear transcription [9]. STAT3 forms dimers when activated by tyrosine kinase signals and translocates to the nucleus to regulate expression of genes by binding to elements within promoters [10]. In vitro and *in mouse* tumor models STAT3 is integrally involved in tumorigenesis, including apoptosis, cell cycle progression, tumor angiogenesis, invasion and metastasis [11–13]. However, there were no studies about *STAT3* gene DNA and mRNA expression changes in invasive or recurrence PAs.

To this purpose, we performed methylation-specific polymerase chain reaction (MS-PCR) to evaluate methylation status of *STAT3* gene promoter and real-time polymerase chain reaction (RT-PCR) to determine the mRNA expression changes in PAs. These epigenetic alterations were then correlated with clinical features of the patients and the invasiveness, recurrence of PAs.

## Methods

### The subject

The study was performed in the Neuroscience Institute, Lithuanian University of Health Sciences between 2010 and 2016. After surgical resection tumor tissues were frozen in nitrogen liquid and the following clinical characteristics were gathered: gender, age at the time of the operation, relapse, tumor size, PA activity, invasiveness, hypersecretion of PRL, GH, IGF-1, ACTH or more than one hormone and diagnoses of Cushing syndrome, acromegaly or prolactinoma.

Study participants comprised of 102 patients (56.86% women and 43.14% men, 60.78% older than 60 years and 39.22% younger than 60 years). Clinical findings showed that the endocrinological features were: 54 functioning and 48 non-functioning adenomas. Functioning adenomas were: 4 GH-secreting adenomas, 2 IGF-1-secreting adenomas, 36 PRL-secreting adenomas, 1 ACTH-secreting adenoma and 11 adenomas secreting more than one hormone. Also, magnetic resonance imaging findings and the Hardy classification, modified by Wilson was used to quantify the sphenoid sinus invasion and suprasellar extension [14]. In connection with the fact that not for all patients were able to receive data, relapse was established in 14 and invasiveness - in 88 patients. From them, it was found 58 invasive and 30 non-invasive PAs. It is important to mention that all PA tumors were macroadenomas.

### Methylation-specific polymerase chain reaction (MS-PCR)

For the DNA methylation studies, 102 frozen PA tissues were used. First, DNA was extracted by 10% “UtraPure™ ”SDS (Invitrogen, USA), proteinase K (Carl Roth® GmbH, Germany), followed by phenol–chloroform (Carl Roth® GmbH, Germany) and 96% ethanol (Stumbras, Lithuania) precipitation. Subsequently, according to the manufacturer’s instructions, genomic DNA was modified with sodium bisulfite using EZ DNA methylation kit™ (Zymo Research, USA) and eluted in 40 μL of nuclease-free water.

The MS-PCR was performed in 15 μl of a mixture containing 10 pmol of each primer (Metabion International AG, Germany), 7.5 μL Maxima® Hot Start PCR Master Mix (ThermoFisher Scientific, USA) with Hot Start Taq DNA polymerase and nuclease-free water. MethPrimer online tool [15] was used to design primers for methylated STAT3 allele: 5′-TTTTGGGTGGTCGAACG-3′ (forward), 5′-AAAAACAACGCCAAACCG-3′ (reverse), resulting in a 222 bp PCR amplicon and for non-methylated allele: 5′-ATTTTTGGGTGGTTGAATG-3′ (forward), 5′-AAAAAAAACAACACCAAACC-3′ (reverse), resulting in a 225 bp PCR amplicon. The reaction was hot started at 95 °C for 5 min and MS-PCR conditions for all of the reactions were as follows: denaturation at 95 °C for 15 s, annealing at 60 °C for 30 s and extension at 72 °C for 15 s, for 38 cycles, and final 5 min extension at 72 °C. In each set of methylation-specific PCR reactions three controls were included: normal human blood lymphocyte DNA treated with bisulfite (non-methylated control), the Bisulfite-Converted Universal Methylated Human DNA Standard (Zymo Research, USA) (methylated control) and nuclease-free water (negative control).

The MS-PCR products were analyzed on a 2% agarose gel with ethidium bromide (10 mg/ml, Invitrogen, USA) by horizontal electrophoresis.

### Quantitative real-time PCR (qRT-PCR)

For the *STAT3* gene mRNA expression analysis, total RNA was extracted from the same set of PA samples (102 samples) that has been used for *STAT3* gene methylation analysis with Trizol reagent (Ambion, Life Technologies, USA), according to the manufacturer’s instructions and stored at −80 °C. RevertAid H Minus M-MuLV Reverse Transcriptase (ThermoFisher Scientific, USA) and random hexamer primers (ThermoFisher Scientific, USA) were used to produce the first-strand cDNA. Total reaction volume was 20 μl. Negative controls without reverse transcriptase were prepared as above.

Quantitative real-time PCR for *STAT3* and *β-actin* was performed in a Real-Time PCR System “Applied Biosystems 7500 Fast”(Applied Biosystems, USA) with SYBR Green chemistry. The qRT-PCR was carried out in a 12 μl of mixture which consisted of 6 μl Maxima SYBR Green/ROX qPCR Master Mix (2X) (ThermoFisher Scientific, USA), 15 ng of the cDNA, nuclease-free water and *STAT3* gene-specific primers that were designed according to the published data [16]: 5′-CATATGCGGCCAGCAAAGAA-3′ (forward), 5′-ATACCTGCTCTGAAGAAACT-3′ (reverse), resulting in a 152 bp PCR amplicon to a total concentration of 0.3 μM. The housekeeping gene *β-actin* with primers designed according to the published data [17]: 5′- AGAGCTACGAGCTGCCTGAC-3′ (forward), 5′-AGCACTGTGTTGGCGTACAG-3′ (reverse), resulting in a 184 bp PCR amplicon to a total concentration of 0.1 μM, was used as an internal control. The PCR amplification cycles were as follows: the denaturation step at 95 °C for 10 min and then 92 °C for 30 s, 60 °C for 30 s and 72 °C for 30 s for 40 cycles and a final step for the generation of a melting curve. Also, *GAPDH* TaqMan assay (Applied Biosystems, Hs02758991_g1, cat. No. 4331182) was used to verify the expression of another endogenous control in PAs. The PCR amplification was performed according to the manufacturer’s protocol. All sample measurements were performed in triplicate. Healthy human brain RNA sample (RHB) “FirstChoice Human Brain Reference RNA” (Ambion, Life Technologies, USA, cat. No. AM6050) was used for standard curve design and the parameters were: for *STAT3*: efficiency 92.68%, R^2^ = 0.99, slope − 3.51; for *β-actin*: efficiency 100.08%, R^2^ = 0.99, slope − 3.32.

Finally, results were presented as 2^-ΔΔCt^ calculations, where ΔΔCt = (Ct,_*STAT3*_ - Ct, _*internal controls*_)_Sample x_ - (Ct,_*STAT3*_ - Ct, _*internal controls*_)_RHB_ [18].

### Statistical analysis

Statistical analyses were conducted with the SPSS Statistics 19 (SPSS Inc., Chicago, IL) and GraphPad Prism 7 (California, USA) software packages. The Mann-Whitney test was used to find the associations between *STAT3* gene mRNA expression and the methylation status, also the clinical factors: gender, age, relapse, Cushing syndrome, acromegaly, prolactinoma, invasiveness, secreting and non-secreting PAs and hormone groups. The associations among *STAT3* gene promoter methylation and clinical characteristics of PA patients were assessed with Chi-square test and *p < 0.05* values were considered statistically significant.

## Results

### Analysis of *STAT3* methylation frequency in patients with PA

**Fig. 1:**
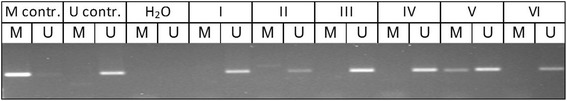
Analysis of *STAT3* gene MS-PCR products on 2% agarose gel. M indicates methylated alleles, U unmethylated alleles. M cont. – positive methylation control (Standard Bisulfite Converted Universal Methylated Human DNA), U cont. – negative methylation control (normal human peripheral lymphocytes), H_2_O – water control, I-VI designate PA samples

*STAT3* gene methylation status was performed in 102 PA samples by the MS-PCR method. The representative chart of MS-PCR products that were separated by horizontal electrophoresis and visualized under UV illumination is shown in Fig. 1. All PA samples were compared with positive and negative controls. Our results showed that *STAT3* gene was methylated in 10.78% (11/102) and non-methylated in 89.22% (91/102) of PA samples. These results indicate that *STAT3* has low methylation status in PAs.

Next, we tried to find out the associations between *STAT3* gene promoter methylation and patient clinical characteristics: gender, age at the time of operation, relapse, PA function, invasiveness and diagnoses of Cushing syndrome, acromegaly or prolactinoma. To this purpose, Chi-square test was performed. However, statistical analysis showed no significant differences (Table 1). In addition, invasive PAs showed low methylation status of *STAT3* gene. Only in 12.07% (7/58) invasive PAs *STAT3* gene was methylated and non-methylated in 87.93% (51/58) cases but with no significance (χ^2^ test, *p* = 0.428).

**Table 1:** Relationship between *STAT3* promoter methylation, patient clinical characteristics and PA invasiveness

	*STAT3* gene methylation			
	Number of patients	M (%)	U (%)	*p*-value
Cases	102	11(10.78)	91(89.22)	
Age (years)				
≤ 60	40	4(10.00)	36(90.00)	0.837
> 60	62	7(11.29)	55(88.71)	
Gender				
Female	58	6(10.34)	52(89.66)	0.870
Male	44	5(11.36)	39(88.64)	
PA function				
Secreting	54	6(11.11)	48(88.89)	0.910
Non-secreting	48	5(10.42)	43(89.58)	
Relapse				
Appear	8	1(12.50)	7(87.50)	0.871
None	94	10(10.64)	84(89.36)	
Prolactinoma				
Appear	36	4(11.11)	32(88.89)	1.000
None	18	2(11.11)	16(88.89)	
Acromegaly				
Appear	9	1(11.11)	8(88.89)	1.000
None	45	5(11.11)	40(88.89)	
Cushing syndrome				
Appear	1	0(0.00)	1(100.00)	0.721
None	53	6(11.32)	47(88.68)	
Hormones				
PRL	36	4(11.11)	32(88.89)	
IGF-1	2	0(0.00)	2(100.00)	
GH	4	2 (50.00)	2(50.00)	
ACTH	1	0(0.00)	1(100.00)	
Multiple	11	0(0.00)	11(100.00)	
Invasiveness				
Invasive	58	7(12.07)	51(87.93)	0.428
Non-invasive	30	2(6.67)	28(93.33)	

*M* methylated, *U* non-methylated, *PRL* prolactin, *IGF-1* insulin-like grow factor 1, *GH* growth hormone, *ACTH* – adrenocorticotropic hormone, multiple – PAs secreting more than one hormone

**Fig. 2:**
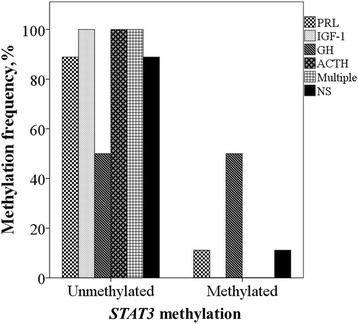
PA hormone distribution in methylated and non-methylated *STAT3* gene groups. PRL – prolactin, IGF-1 – insulin-like grow factor 1, GH – growth hormone, ACTH – adrenocorticotropic hormone, multiple – PAs secreting more than one hormone, NS – non-secreting PAs

To further analysis we wanted to find out in which hormonally active PAs *STAT3* gene promoter might be strongly methylated. Thus, the hormone distribution of PAs in methylated and non-methylated *STAT3* gene promoter groups was performed. The analysis showed that with methylated *STAT3* promoter, the quantities of PRL (4/36), IGF-1 (0/2), ACTH (0/1) and more than one hormone secreting PAs (0/11) hypersecretion were highly reduced as well as the non-secreting PAs (5/48) (Fig. 2). Nevertheless, the amount of GH hypersecretion was the same in methylated and non-methylated gene groups (2/4). However, the results were not statistically significant (Table 1).

### Analysis of *STAT3* mRNA expression in patients with PA

To investigate the *STAT3* mRNA expression changes in PAs, the qRT-PCR method was used. The values were normalized with two internal controls: *GAPDH* and *β-actin*. *STAT3* mRNA expression was determined in 102 PA samples. It was found that in 13 cases there were no *STAT3* mRNA expression. To compare the *STAT3* mRNA expression in methylated and non-methylated gene groups, Mann-Whitney test was used. The analysis showed no significant differences of *STAT3* mRNA expression between the group of methylated *STAT3* (10/89 tumors) and the group of non-methylated *STAT3* gene (79/89 tumors, *p* = 0.795).

**Fig. 3:**
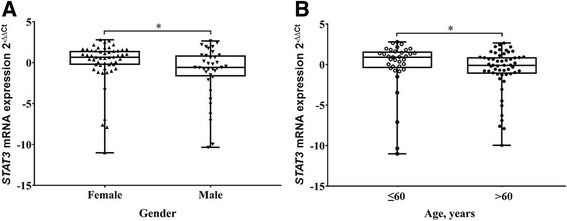
*STAT3* mRNA expression associations with patient characteristics. **a** Box plots of *STAT3* mRNA expression association with gender. **b** Box plots of *STAT3* mRNA expression association with patient age. The line inside box represents the median, the lower and upper edges of the boxes represent the 25th and 75th percentiles, respectively, and upper and lower lines outside the boxes represent minimum and maximum values (error bars). The Mann-Whitney test showed, that males and patient group older than 60 years were significantly associated with reduced *STAT3* mRNA expression (*p* < 0.05)

Next, we assessed the associations between *STAT3* mRNA expression and patient clinical characteristics. We found that gene mRNA expression was lower with males (36/89 cases) than females (53/89 cases, Mann-Whitney test, *p* = 0.025) and with patients older than 60 years (54/89 cases, Mann-Whitney test, *p* = 0.047, Fig. 3). However, there were no statistical differences with presence or absence of repeated surgery, invasiveness, secreting or non-secreting PAs, the diagnosis of acromegaly, prolactinoma or Cushing syndrome (Mann-Whitney test, *p* = 0.272, *p* = 0.798, *p* = 0.812, *p* = 0.935, *p* = 0.312 and *p* = 0.400, respectively).

**Fig. 4:**
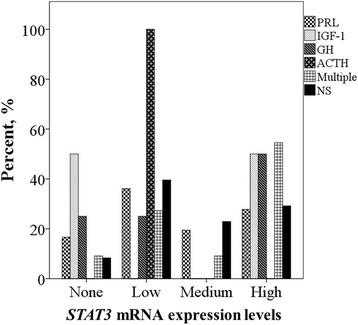
PA hormone distribution in *STAT3* mRNA expression levels. PRL – prolactin, IGF-1 – insulin-like grow factor 1, GH – growth hormone, ACTH – adrenocorticotropic hormone, multiple – PAs secreting more than one hormone, NS – non-secreting PAs

For further investigation *STAT3* gene mRNA expression values were categorized into four levels. Values 0.5-fold lower than the gene mRNA expression median were indicated as “low” expression level (*N* = 37), expression values 0.5-fold higher - “high” level (*N* = 33), values ranging in between “low” and “high” were referred to as “medium” mRNA expression level (*N* = 19). In 13 samples there were no *STAT3* mRNA expression. Then we analyzed PRL, IGF-1, GH, ACTH, more than one hormone and non-secreting PAs distribution in these expression levels. As was shown in Fig. 4, in most cases the hypersecretion of PRL (13/36), ACTH hormone (1/1) and the highest amount of non-secreting PAs (19/48) were determined at “low”*STAT3* mRNA expression level (χ^2^ test, *p* = 0.321, *p* = 0.565 and *p* = 0.417, respectively). Similarly, GH (2/4) and more than one hormone hypersecretion (6/11) - at “high”*STAT3* mRNA expression level (χ^2^ test, *p* = 0.758 and *p* = 0.493, respectively). The IGF-1 hormone hypersecretion (1/2) mostly occured at “high”*STAT3* mRNA expression level and with no gene expression (χ^2^ test, *p* = 0.473).

**Fig. 5:**
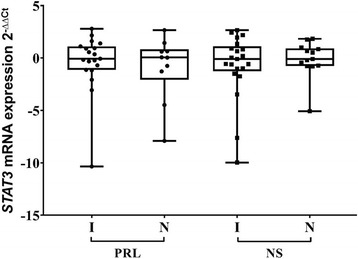
*STAT3* mRNA expression changes in invasive and non-invasive PRL secreting and non-secreting PAs. The horizontal bars represent the mean values with 95% confidence interval, the spots represent the amount of samples. PRL – prolactin, NS – non-secreting PAs

In our last series of result analysis, we took the biggest group of hormonally active PAs (PRL-secreting PAs) and non-secreting PAs and asked if *STAT3* mRNA expression is associated with PA invasiveness (Fig. 5). However, Mann-Whitney test did not reveal significant differences in *STAT3* mRNA expression median values (*p* = 0.769 and *p* = 0.753, respectively).

## Discussion

Pituitary adenoma is a benign brain tumor that could invade the sphenoid, cavernous sinus, or dura mater and some of them are described as aggressive, with a high proliferation rate and short postoperative time to recurrence [1, 19]. Therefore, an epigenetic marker to predict a greater malignant potential of PAs still remains to be established. *STAT3* gene may be a promising target because it is aberrantly activated in a wide variety of human cancers and plays crucial roles in tumor cell proliferation, survival, invasion and tumor-promoting inflammation [20–26].

We conducted this study to examine the prognostic significance of *STAT3* gene promoter methylation and mRNA expression in 102 PA patients and to determine the associations with patient clinical data. We determined 10.78% (11/102) *STAT3* promoter methylation frequency. Also, in our study invasive PAs showed low *STAT3* methylation status. Only in 12.07% (7/58) invasive PAs, *STAT3* promoter was methylated (Table 1). We also demonstrated that PRL, IGF-1, ACTH and more than one hormone hypersecretion mostly occur with non-methylated *STAT3* gene (Fig. 2). Low *STAT3* gene methylation status indicates that this gene is little suppressed in PAs and might be constantly activated. However, mechanisms related to this are still unknown. Numerous studies showed the activation of STAT3 in a wide variety of tumors [20–26] and the proposed role for STAT3 in tumor formation or progression is based on the link between its activation and transformation [10]. For example, Morikawa T et al. (2011) found that individuals with STAT3-activated colorectal cancers experience a poorer prognosis, suggesting a potential tumor-promoting role of p-STAT3 expression as a prognostic biomarker [20]. Increased expression of phosphorylated STAT3 and JAK/STAT activation in glioma samples has also been correlated with significantly shorter overall survival and has been associated with more aggressive tumors, indicating that STAT3 is a key contributor to glioma pathogenesis by mediating cell survival, growth, and proliferation [21–23]. Moreover, in breast carcinoma cells, the STAT3 activation has been correlated with cell proliferation [24], in colon cancer STAT3 has been shown to be overexpressed [25], as well as in high-grade prostate cancer patients [26]. In these mentioned studies, STAT3 has been described as an oncogene, however, there are some other publications in which STAT3 has been shown to be a tumor suppressor. For example, in colorectal carcinoma biopsies STAT3 overexpression has been associated with an improvement in median survival of about 30 months [27]. French patient cohort by Monnien et al. (2010) provided evidence for a association of phosphorylated STAT3 appearance with prolonged survival of rectal cancer patients [28]. Therefore, STAT3 can act as an oncogene or a tumor suppressor, depending on tumor tissue. In our results, there were no other associations between *STAT3* gene promoter methylation, mRNA expression and PA invasiveness, recurrence or patient clinical characteristics. Only a gender of male and patient group older than 60 years were significantly associated with reduced *STAT3* mRNA expression (Mann-Whitney test, *p* = 0.025, *p* = 0.047, respectively). However, the mechanism of *STAT3* expression changes in PA proliferation and invasion has not been reported yet, making it necessary to further research on the pro- and anti-tumorigenic effects of *STAT3* gene activation.

It is important to mention, that Zhou et al. (2015) made a research of STAT3 expression in somatotroph adenomas. They found that STAT3 expression was significantly enhanced in somatotroph adenomas (67% ± 5%) as compared with non-secreting pituitary tumor expression (unpaired t test, *P* < 0.001) [29]. Also, they showed that GH induces somatotroph cell STAT3 phosphorylation and nuclear translocation. Based on these results, Zhou et al. (2015) postulated that GH participates in a positive autocrine or paracrine feedback for STAT3 induction and supported the hypothesis that blocking STAT3 suppresses somatotroph tumor growth and inhibits GH hypersecretion [29]. Our results also showed a tendency that the highest amount of GH was determined at “high” *STAT3* mRNA expression level (Fig. 4), making it necessary for further investigation.

## Conclusions

In conclusion, this is the first study that has demonstrated the *STAT3* gene promoter methylation and mRNA expression in 102 PA samples and analyzed the relationships between *STAT3* gene epigenetic changes and patient clinical characteristics: age at the time of operation, gender, diagnoses of prolactinoma, acromegaly or Cushing syndrome, PA activity, recurrence and invasiveness. Our data have revealed that *STAT3* gene mRNA expression is significantly lower with male gender and patients older than 60 years. However, no more significant results were found. To this purpose, our designed *STAT3* gene promoter methylation analysis and mRNA expression cannot be considered as a prognostic marker in PAs or further examination including new methylation sites investigation for *STAT3* methylation-specific PCR is required to estimate the influence of epigenetic changes in PAs.

## Abbreviations

ACTH: Adrenocorticotropic hormone
GH: Growth hormone
IGF-1: Insulin-like growth factor 1
MS-PCR: Methylation-specific polymerase chain reaction
PA: Pituitary adenoma
PRL: Prolactin
qRT-PCR: Quantitative real-time polymerase chain reaction
RHB: Human brain reference RNA
STAT3: Signal transducer and activator of transcription 3
